# Critical Dynamics in Genetic Regulatory Networks: Examples from Four Kingdoms

**DOI:** 10.1371/journal.pone.0002456

**Published:** 2008-06-18

**Authors:** Enrique Balleza, Elena R. Alvarez-Buylla, Alvaro Chaos, Stuart Kauffman, Ilya Shmulevich, Maximino Aldana

**Affiliations:** 1 Instituto de Ciencias Físicas, Universidad Nacional Autónoma de México, Cuernavaca, Morelos, México; 2 Instituto de Ecología, Universidad Nacional Autónoma de México, San Nicolás de Los Garza, México; 3 Institute of Biocomplexity and Informatics, University of Calgary, Calgary, Alberta, Canada; 4 Institute for Systems Biology, Seattle, Washington, United States of America; Children's Hospital Boston, United States of America

## Abstract

The coordinated expression of the different genes in an organism is essential to sustain functionality under the random external perturbations to which the organism might be subjected. To cope with such external variability, the global dynamics of the genetic network must possess two central properties. (a) It must be robust enough as to guarantee stability under a broad range of external conditions, and (b) it must be flexible enough to recognize and integrate specific external signals that may help the organism to change and adapt to different environments. This compromise between robustness and adaptability has been observed in dynamical systems operating at the brink of a phase transition between order and chaos. Such systems are termed *critical*. Thus, criticality, a precise, measurable, and well characterized property of dynamical systems, makes it possible for robustness and adaptability to coexist in living organisms. In this work we investigate the dynamical properties of the gene transcription networks reported for *S. cerevisiae*, *E. coli*, and *B. subtilis*, as well as the network of segment polarity genes of *D. melanogaster*, and the network of flower development of *A. thaliana*. We use hundreds of microarray experiments to infer the nature of the regulatory interactions among genes, and implement these data into the Boolean models of the genetic networks. Our results show that, to the best of the current experimental data available, the five networks under study indeed operate close to criticality. The generality of this result suggests that criticality at the genetic level might constitute a fundamental evolutionary mechanism that generates the great diversity of dynamically robust living forms that we observe around us.

## Introduction

There is evidence that many complex dynamical systems found in nature are critical; namely, they operate close to a phase transition between two different dynamical regimes [1]. Avalanches [2], atmospheric phenomena [3], [4], financial markets [5], [6], earthquakes [7], [8], granular matter [9], and the brain [10]–[12], are typical examples. Critical systems exhibit remarkable properties which would be difficult to explain without the assumption of criticality. For instance, they can integrate, process and transfer information faster and more reliably than non critical systems [13]. Or they can detect and respond to external stimuli whose intensities span several orders of magnitude, like the brain [11]. These remarkable properties are mainly a consequence of the long-range correlations that emerge close to the critical point, producing collective behaviors and coordinated responses of the entire system. Thus, criticality confers on the system the ability to collectively respond and adapt to an often rapidly changing environment.

In the context of genetic regulatory networks (GRN), which are recognized as the main component in charge of cellular control [14], recent theoretical studies have shown that robustness and adaptability, two central properties of living organisms [15]–[20], exist simultaneously with the highest probability only in GRN operating at or close to criticality [21]. Thus, criticality is a property that can help us understand how the coordinate expression of the different genes in an organism is achieved under external perturbations, either to sustain cell functionality or to generate new phenotypes in order for the organisms to change and adapt to new environments [15]–[21]. Therefore, it is important to determine whether the GRN of real organisms are dynamically critical. Although some attempts have recently been made in order to answer this question [22]–[25], the definite answer has remained elusive for several decades. Here we present direct evidence that the GRN of five different organisms indeed exhibit critical dynamics. We do so by simulating in the computer the avalanche of perturbations in the gene expression profile of the genetic networks of these organisms. This allows us to compute the Derrida mapping *M*(*x*) for the five networks under consideration [26]. We will formally introduce the Derrida map in a further section. For the time being, it suffices to say that *M*(*x*) relates the size *x*(*t*) of the perturbation avalanche at time *t*, with the size *x*(*t*+1) of the avalanche at the next time step *t*+1. In other words: *x*(*t*+1) = *M*(*x*(*t*)). Therefore, *M*(*x*) contains all the information of the perturbation dynamics and can be used to directly measure the dynamical regime in which the network operates. Using this technique, we show that the dynamics of these avalanches are critical within numerical accuracy for the five different organisms studied.

However, in computing *M*(*x*) for the large networks of *E. coli*, *S. cerevisiae* and *B. subtilis*, we face the problem that the overwhelming majority of the regulatory functions (also termed *regulatory phrases*) that determine the *combined* effect of the regulators on their target genes are unknown. To circumvent this difficulty, we used random functions to model the dynamics of these networks. Although random, these functions were constructed in accordance with the fraction of positive regulatory phrases inferred from real gene expression profiles. Thus, the internal structure of the regulatory functions that we used for *E. coli*, *S. cerevisiae* and *B. subtilis* is statistically consistent with the one observed in microarray experiments.

Another important aspect that determines the dynamical regime in which the network operates (ordered, critical or chaotic) is the fraction of canalizing functions [27]–[31], which will be defined in a further section. Intuitively, these functions take into account the existence of dominant regulators such that, when present, override the effect of the other regulators. From the microarray experiments that we analyze it was not feasible to infer the fraction of canalizing functions present in the regulatory networks of *E. coli*, *S. cerevisiae* and *B. subtilis*. However, for these networks we varied in our simulations the fraction of canalizing functions around the statistically expected values. Interestingly, we observe a significant robustness of the critical dynamics under the addition or elimination of canalizing functions. This suggests that the critical behavior observed in the dynamics of the genetic networks of the organisms under study, is mainly produced by the network architecture rather than by the specific nature of the regulatory functions.

In the sections that follow we first present the Boolean network model that we use to implement the dynamics of the genetic networks, and the well known mean-field results that predict the existence of a phase transition from ordered to chaotic dynamics in this model. Then, we go beyond the mean-field theory by implementing the Boolean approach in the networks of real organisms, and show that in all the cases the Derrida map *M*(*x*) is consistent with critical dynamics. We then analyze how this map changes under the addition and removal of canalizing functions. In the last section we summarize and discuss our results.

## Results

### Boolean Models of Genetic Networks

Several models have been proposed to analyze the dynamics of GRN [32]–[35]. Although the details of the dynamics might change from one description (e.g. continuous) to another (e.g. discrete), we expect the general properties of the dynamics, such as criticality, to be model independent. In fact, recent work shows that continuous and discrete descriptions of GRN exhibit similar dynamical properties under very general conditions [36]. Here we use the Boolean approach [37]–[39], in which every gene is represented by a discrete variable *g* that can take two values: *g* = 1 if the gene is expressed and *g* = 0 otherwise. The genome is thus represented by a set of *N* binary variables, *g_1_*, *g_2_*, …, *g_N_*. The expression of each gene *g_n_* changes in time according to the equation
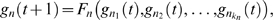
(1)where 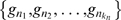 are the *k_n_* regulators of *g_n_*, and *F_n_* is a Boolean function (also known as a logical rule), which is constructed according to the inhibitory or activatory nature of the regulators. The value acquired by the Boolean function for each configuration of the regulators is termed a *regulatory phrase*. For instance, if *F*(*g*_1_, *g*_2_) is a function of the two regulators *g*_1_ and *g*_2_ such that *F*(1,1) = 1, *F*(1,0) = 1, *F*(0,1) = 0, and *F*(0,0) = 0, then this function consists of the four regulatory phrases {1,1}→1, {1,0}→1, {0,1}→0, and {0,0}→0. We will refer to the regulatory phrases for which *F* = 1 as *activatory*, and those for which *F* = 0 as *inhibitory*. The fraction *p* of activatory phrases in the entire network, called the *gene expression probability*, is an important parameter that controls the dynamical regime in which the network operates (i.e. ordered, critical or chaotic). Recent work shows that the Boolean approach does capture the main aspects of the gene regulation dynamics, for it is able to reproduce gene expression patterns observed experimentally for several organisms [35], [40]–[43].

### Phase Transition in the Boolean Network Model

In this section we present the mean-field theory results that show the existence of a dynamical phase transition from ordered to chaotic dynamics in the Boolean network model [26], [38], [44]. This allows us to introduce the tools that we use to characterize the dynamical regime in which the network operates. Although the phase transition was first obtained within the context of the mean-field approximation, we will show in the next section the remarkable result that the phase transition also occurs, almost identically, in networks with realistic topologies, for which the mean-field assumption does not necessarily apply. The phase transition is characterized by the temporal evolution of the Hamming distance *x*(*t*) between two different dynamical trajectories produced by two slightly different initial conditions. From a biological point of view, the Hamming distance *x*(*t*) is the average normalized size at time *t* of the avalanche of perturbations in the gene expression profile, produced by the perturbation (e.g. gene knockout or gene over expression) of a small fraction *x*(0) of genes at time *t* = 0. The temporal evolution of *x*(*t*) is given by a dynamical mapping *x*(*t*+1) = *M*(*x*(*t*)) which relates the size of the perturbation avalanche at two consecutive time steps [26], [38], [44]. Given an initial perturbation *x*(0) at time *t* = 0, successive iterations of this mapping will eventually converge to a stable fixed point 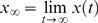, which is the final size of the perturbation avalanche. The value *x*_∞_ is the order parameter that characterizes the dynamical regime in which the network operates. Thus, if *x*_∞_ = 0 (ordered regime), all the initial perturbations die out over time. On the contrary, if *x*_∞_>0 (chaotic regime), the initial perturbation of even a small fraction of genes propagates across the entire system, finally altering the expression of a finite fraction *x*_∞_ of genes in the genome. It turns out that *M*(*x*) is a continuous convex monotonically increasing function of *x* (in the mean field theory *M*(*x*) is a polynomial), with the property that *M*(0) = 0 and *M*(1)<1. Therefore, there is only one parameter that controls the phase transition, the so-called average network sensitivity *S*, which is given by *S* = [*dM*(*x*)/*dx*]*_x_*_ = 0_. If *S*<1 then the only fixed point is *x*_∞_ = 0, whereas if *S*>1 then *x*_∞_>0. The phase transition occurs at *S* = 1, for which the fixed point *x*_∞_ = 0 is only marginally stable. In general, *S* depends on *p* and on the topology of the network. The dynamical mapping *M*(*x*) contains all the information about the dynamical regime in which the network operates. This is true even if *M*(*x*) cannot be obtained through a mean-field computation, which is the case for real networks. In the Supporting Information (Text S1) we provide a Java applet with the animation of the perturbation dynamics in networks with homogeneous random topology.

### Beyond the mean-field theory: Existence of the Phase Transitions in Networks with Realistic Topologies

**Figure 1:**
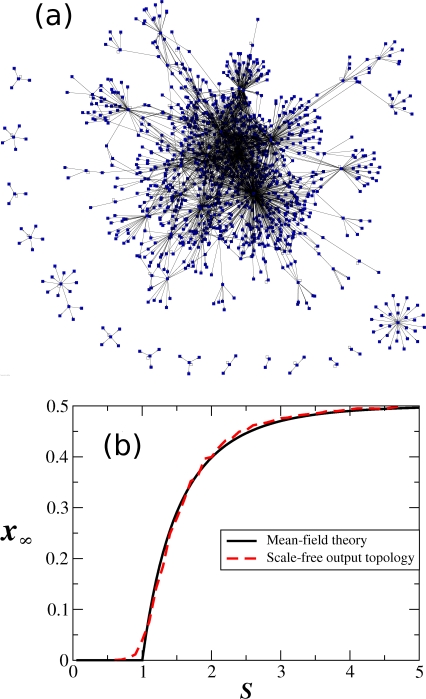
Order-chaos phase transition in Boolean networks with realistic topologies. (a) Graphic representation of the gene transcription network of *E. coli K-12*. For this network the probability for a given gene to have *K* regulators is *P*(*K*) = *e* ^−*z*^ *z^K^*/*K*! (Poisson input topology), whereas the probability to be a regulator to *n* other genes is *P*(*n*) = *Cn* ^−γ^ (scale-free output topology). (b) Phase transition diagram showing *x*_∞_ as a function of *S*. The solid line in black is the theoretical result predicted by the mean-field theory. The dashed line in red was computed numerically for randomly constructed Boolean networks with *N* = 1000, Poisson input topology and scale-free output topology. The Boolean functions were randomly generated with a probability of gene expression *p* = 0.5. Remarkably, the phase transition predicted by the mean field theory is identical to the one obtained for random Boolean networks with topologies statistically equivalent to the real ones.

The mean-field theory that predicts the existence of the phase transition controlled by the average network sensitivity *S*, is based on the assumption that all the genes in the network are statistically independent and statistically equivalent [26], [44]. However, this assumption is certainly not true for real GRN, due to the existence of global regulators which correlate the expression of a large fraction of genes. Indeed, recent large-scale analysis [21] of the transcription regulatory networks of *E. coli*, *S. cerevisiae*, and *B. subtilis* indicate that these networks exhibit a Poisson-like input topology and a scale-free output topology (see Figure 1a). The output scale-free topology correlates the expression of a large fraction of the genes, and the assumption of statistical independence is not longer satisfied. Therefore, we do not expect the mean-field theory to be applicable for real networks because of their topological characteristics. Nonetheless, the phase transition predicted by the mean-field theory is identical to the one observed in randomly constructed Boolean networks with topologies statistically equivalent to the ones observed in real GRN. This is shown for the first time in Figure 1b. This result is quite remarkable, for we know that in many other systems the phase transition strongly depends on the network topology, and can even disappear for topologies that induce strong correlations between the elements (such as the scale-free topology, [45]).

### Critical Dynamics in Real Genetic Networks

To determine the dynamical regime in which the genetic network of a real organism operates, we have to compute the dynamical mapping *M*(*x*) directly from experimental data, without any mean-field assumptions. The actual form of *M*(*x*) depends on both the network topology and the particular set of Boolean functions. We computed numerically *M*(*x*) for five genetic networks: The network of flower morphogenesis in *A. thaliana* (15 genes; [41]); the network of segment polarity genes in *D. melanogaster* (60 genes; [42]); and the gene transcription networks of *E. coli* (1481 genes; [46]), *S. cerevisiae* (3459 genes; [47]), and *B. subtillis* (830 genes; [48]). In the first two cases the Boolean functions are already known. However, the overwhelming majority of regulatory phrases for the gene transcription networks of *E. coli*, *S. cerevisiae* and *B. subtilis* are still unknown. Due to this lack of information, to implement the Boolean dynamics on the GRN of these three organisms we used biased random Boolean functions generated with a gene expression probability *p* inferred from microarray experiments. (In the next section we show that the map *M*(x) does not change significantly for networks with a large fraction of canalizing functions.) *Given* the network topology, *p* can be estimated from microarray experiments by standard Bayessian parametric inference with two states (see Methods). Using 223 microarrays to sample the gene expression space in *S. cerevisiae* [49], 152 microarrays for *E. coli* [49]–[51], and 69 microarrays for *B. subtillis* [52], we inferred several regulatory phrases for each of these organisms. All the experiments used for this analysis are listed in the Supporting Information accompanying this article (Text S1). We only used regulatory phrases for which the average of the *a posteriori* probability distribution was greater than 70% (activatory phrase) or smaller than 30% (inhibitory phrase). The inference technique is explained below in the Materials and Methods section. With this technique, we obtained the following gene expression probabilities: *p* = 0.576±0.038 for *E. coli* (from 264 inferred phrases), *p* = 0.495±0.055 for *S. cerevisiae* (from 196 phrases), and *p* = 0.0531±0.035 for *B. subtilis* (from 307 phrases). We then constructed random Boolean functions with internal bias given by these probabilities.

**Figure 2:**
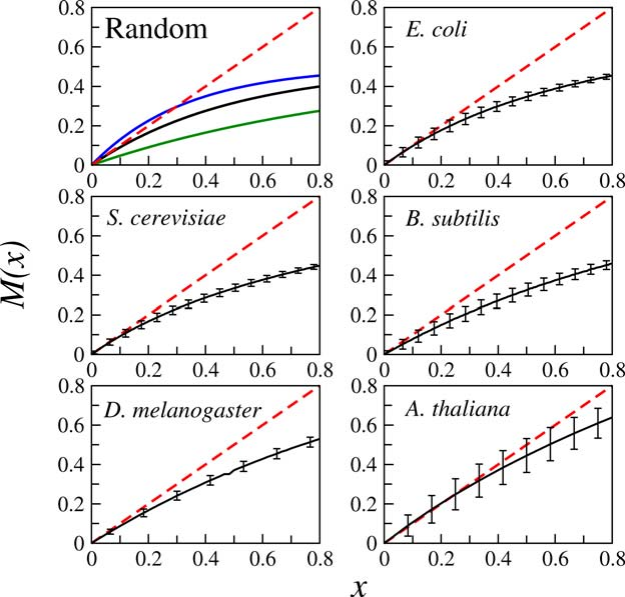
Critical dynamics in networks of real organisms. Derrida curves exhibiting critical dynamical behavior in the genetic networks of five different organisms spanning four kingdoms of life: *E. coli* [46], *S. cerevisiae* [47], and *B. subtilis* [48], *D. melanogaster* [42] and *A. thaliana* [41]. Each point in the Derrida curve is the average over 20000 initial perturbations and the error bars indicate one standard deviation around this average. Additionally, the error bars in the curves of *E. coli*, *B. subtilis* and *S. cerevisiae* incorporate the uncertainty in the estimation of the *p* bias inferred from the microarray experiments. For comparison we show in the upper left corner three Derrida curves of randomly constructed networks with Poisson input topology operating in three dynamical regimes: Ordered (green), critical (black) and chaotic (blue). Note that criticality is characterized by the tangency of the Derrida curve to the identity close to *x* = 0.

We report in Figure 2 the Derrida curves (i.e. the graphs of *M*(*x*)) for the five genetic networks under consideration. It is apparent from this figure that in all five cases the Derrida curves arrive almost tangent to the identity close to the origin. The above is consistent with critical behavior at the genetic level. A polynomial regression analysis allows us to estimate the average network sensitivity *S* = [*dM*(*x*)/*dx*]*_x_*_ = 0_ by computing the slope at the origin of the best-fit polynomial with a degree equal to the maximum number of regulators per gene in each case (this is the polynomial predicted by the mean-field theory). In all five cases the Regression Sum of Squares is below 10^−4^, and the average network sensitivities obtained in this way are: *S* = 1.028 for *E. coli*; *S* = 1.036 for *S. cerevisiae*; *S* = 0.826 for *B. subtilis*; *S* = 0.914 for *D. melanogaster*; and *S* = 1.127 for *A. thaliana*. Within numerical accuracy, these sensitivities show that the dynamics of these networks are very close to criticality. Note that this is particularly true for the two largest and most complete networks of *E. coli* and *S. cerevisiae*.

### Criticality and its robustness revealed by varying the degree of canalization

We have used random Boolean functions in our simulations because the overwhelming majority of the logical rules for *E. coli*, *S. cerevisiae* and *B. subtilis*, are still unknown. The caveat is that real biological functions are not random. It has been pointed out that canalizing functions are more realistic from a biological point of view [27], [28]. A formal definition of canalizing functions can be found in Ref. [31]. Here, it suffices to define a canalizing function as follows. Let *F*(*g*_1_, *g*_2_, …, *g_k_*) be a Boolean function of *k* arguments. We will say that *F* is canalizing on one of its arguments *g_i_*, if the value of *F* is determined by fixing the value of *g_i_* either to 0 or to 1. To illustrate this concept, Table 1 shows a function *F*(*g*_1_, *g*_2_, *g*_3_) that depends on three arguments. In this example, *F* = 0 whenever *g*_2_ = 1, regardless of the values of *g*_1_ and *g*_3_. Therefore, *F* is canalizing on *g*_2_. The biological significance of canalizing functions is based on the existence of dominant regulators. Thus, in the example shown in Table 1, *g*_2_ could represent a dominant repressor (like *crp* in *E. coli*) which, when present, blocks the transcription of the target gene regardless of the presence or absence of the activators.

**Table 1:** Example of canalizing function.

*g*_1_	*g*_2_	*g*_3_	*F*(*g*_1_, *g*_2_, *g*_3_)
1	1	1	0
1	1	0	0
1	0	1	1
1	0	0	1
0	1	1	0
0	1	0	0
0	0	1	1
0	0	0	0

Example of a Boolean function of three arguments that is canalizing on one of them. Note that *F* = 0 whenever *g*_2_ = 1, regardless of the values of the other two arguments.

It is known that the amount of canalizing functions in the system can change the dynamical regime in which the network operates [29], [30]. Therefore, it is important to determine if the dynamics of the large networks of *E. coli*, *S. cerevisiae* and *B. subtilis* are still critical when more realistic Boolean functions are used. However, from the microarray experiments that we analyzed it is impossible to know the fraction of canalizing functions present in these organisms, or if such functions have one, two or more canalizing inputs. For instance, according to the *regulonDB*, which gives the topology of the transcription regulatory network of *E. coli* [46], there are at least 817 genes with two or more regulators. These 817 genes are all regulated by a subset of 160 genes. Therefore, to determine whether or not the Boolean functions associated with these genes are canalizing, one would have to analyze microarray experiments probing at least 2^160^ different configurations for these genes. This is impossible for many reasons, but mainly because not all the 2^160^ configurations of the regulators are biologically attainable. (Consider for instance the configuration 00000….00 in which all the regulators are “off”, or the configuration 11111…11 in which all the regulators are “on”. Clearly, these two configurations are not attainable under any biological condition—without killing the organism.) Therefore, the 152 microarrays used to sample the gene expression space in *E. coli* represent a very tiny fraction of all the possible configurations necessary to determine the whole set of Boolean functions. However, these 152 experiments are absolutely relevant because they represent the *observable and biologically attainable* gene expression configurations of the organism. Thus, it might be irrelevant if the whole Boolean function of a given gene is canalizing because neither us nor the organism are sampling its whole set of (mathematically possible) configurations. For this reason, we do believe that the important quantity is the *observed* gene expression probability for the biologically attainable configurations.

Nonetheless, Random Boolean functions already contain canalizing functions. Table 2 gives the probability *P_c_*(*K*) for a randomly generated Boolean function with *K* inputs to be canalizing on at least one of its inputs (data taken from [31]). As we can see from Table 2, the probability for a randomly generated Boolean function to be canalizing is high for *K* = 1, 2 and 3 (Boolean functions with *K* = 1 are canalizing, by definition). On the other hand, Table 3 shows the distribution *P_E_*(*K*) of the number of genes with *K* inputs in the genetic network of *E. coli*, according to the last update of the regulonDB [46].

**Table 2:** Fraction of canalizing functions.

*K*	*P_c_*(*K*)
1	1
2	0.875
3	0.468
4	0.0536
5	3.0×10^−4^
6	5.5×10^−9^
7	1.6×10^−18^
8	6.7×10^−38^
9	3.1×10^−76^
10	2.9×10^−153^

Probability *P_c_*(*K*) for a randomly generated Boolean function with *K* inputs to be canalizing. Data taken from [31].

**Table 3:** Distribution of regulators per gene in E. coli.

*K*	*P_E_*(*K*)
0	50
1	615
2	347
3	240
4	100
5	85
6	22
7	11
8	8
9	3
10	1

Distribution *P_E_*(*K*) of the number of genes with *K* regulators in the *E. coli* network according to the last update of the regulonDB [46].

From Tables 2 and 3 we obtain that, if the Boolean functions for *E. coli* are generated at random, just by chance about 
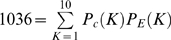
 functions out of 1482 (70%) would be canalizing on at least one input. If we do not consider the 615 genes with only one regulator (because for such genes the Boolean function is trivially canalizing), and the 50 genes with no inputs, then there are 817 genes with two or more regulators. From the data listed in Tables 2 and 3 one obtains that, for the genes with *K*≥2, about 
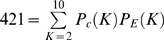
 out of 817 of the randomly generated functions would be canalizing just by chance. It is important to note that these 421 canalizing functions mostly come from the genes with *K* = 2 and *K* = 3. Thus, combining the results presented in Tables 2 and 3 we obtain that the fraction *f_c_* of canalizing functions present just by chance for the genes with *K*≥2 is given by *f_c_*≈421/817≈0.51.

Therefore, in the simulations with random Boolean functions there is already a large fraction of canalizing functions. To further investigate the effect that canalizing functions might have on the dynamics of the genetic networks, we have added and removed canalizing functions to and from the ones already present just by chance. Our results indicate that the critical behavior observed in the genetic dynamics still exists even when the fraction of canalizing functions substantially deviates from the value expected for fully random functions.

**Figure 3:**
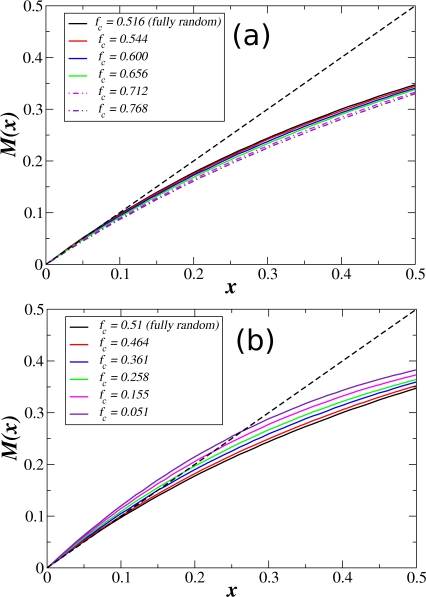
Critical dynamics in *E. coli* using canalizing functions. Derrida map *M*(*x*) for the *E. coli* network with different fractions *f_c_* of canalizing functions. To compute *f_c_* we took into consideration only the genes for which *K*≥2. For fully random Boolean functions *f_c_* = 0.51. (a) We increased the value of *f_c_* by adding canalizing functions to the 230 genes with *K*≥4. Note that *M*(*x*) remains critical even for *f_c_*≈0.66, but it starts to deviate from criticality towards the ordered region for *f_c_*>0.7 (dotted dashed curves). (b) We decreased the value of *f_c_* by removing canalizing functions from the 421 genes which originally are regulated by canalizing functions just by chance (most of them with *K* = 2 and *K* = 3). In this case the curve deviates from criticality towards the chaotic region only for *f_c_*<0.4. Each point in the curves is the average over 5000 initial conditions randomly chosen.

Figure 3 shows the map *M*(*x*) for the *E. coli* network for several values of the fraction *f_c_* of genes with *K*≥2 regulated by canalizing functions. Figure 3a corresponds to the case in which there are more canalizing functions than the ones present just by chance, whereas Figure 3b shows the opposite case in which there are less canalizing functions. To compute *f_c_* we have ignored the genes with only one regulator, taking into account only the 817 genes with two or more regulators. We have already mentioned that in the *E. coli* network, *f_c_* = 0.51 for fully random Boolean functions. Additionally, from Table 2 we also see that for *K* = 2 and *K* = 3 the probability *P_c_*(*K*) for a random Boolean function to be canalizing is relatively high (*P_c_*(2) = 0.875 and *P_c_*(3) = 0.468), whereas for *K*≥4 the probability is extremely low. Therefore, what we did to increase the value of *f_c_* was to add canalizing functions only to the genes with *K*≥4 in such a way as to preserve the overall value *p* = 0.576±0.038 of the gene expression probability observed in microarray experiments. There are 230 genes with *K*≥4, and we can consider that none of them are regulated by canalizing functions just by chance (the probability is very low). Therefore, to increase *f_c_* we *forced* a fraction *q* of these 230 genes to be regulated by canalizing functions. In Figure 3a we present the Derrida maps for *q* = 0.1, 0.3, 0.5, 0.7 and 0.9. When considering the other 421 genes already regulated by canalizing functions just by chance, these values of *q* correspond to *f_c_* = 0.544, 0.600, 0.656, 0.712, and 0.768, respectively. (We computed *f_c_* as *f_c_* = (421+230×*q*)/816). As we can see from Figure 3a, the map *M*(*x*) is practically the same even for *f_c_* = 0.656, namely, even when two thirds of the genes with two or more inputs are regulated by canalizing functions. Only for *f_c_*>0.7 significant deviations from criticality are observed (dotted-dashed curves in Figure 3).

Analogously, to decrease the value of *f_c_* we removed a fraction *q* of the canalizing functions from the 421 genes which originally are regulated by canalizing functions just by chance. In other words, we forced a fraction *q* of these 421 genes to be regulated by non-canalizing functions. In this case, *f_c_* is given by *f_c_* = 421(1−*q*). Figure 3b shows the Derrida maps for *q* = 0.1, 0.3, 0.5, 0.7 and 0.9.

From Figure 3 it is clear that the Derrida map is less robust to the removal than to the addition of canalizing functions. However, in the region 0.4≤*f_c_*≤0.66 the Derrida map does not seem to change substantially.

**Figure 4:**
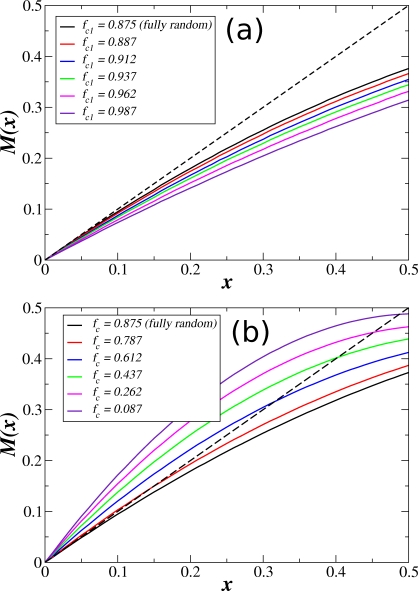
Dynamics of random networks using canalizing functions. Derrida map *M*(*x*) for homogeneous random networks with *N* = 1481, *K* = 2, *p* = 0.5 and different fractions *f_c_* of canalizing functions. For fully random functions the networks operate in the critical regime and *f_c_* = 0.875. Panel (a) shows the results when more canalizing functions are added on top of the ones already present just by chance, whereas panel (b) corresponds to removing canalizing functions from the ones already present. Note that the addition or removal of even a small fraction of canalizing functions in homogeneous random networks has a considerably bigger impact on the dynamics than for the *E. coli* network. Each point in the curves is the average over 5000 initial conditions randomly chosen.

To indicate the significance of the above results we present in Figure 4 the map *M*(*x*) for homogeneous random networks with different fractions *f_c_* of canalizing functions. In these networks each gene has exactly *K* = 2 regulators chosen randomly from anywhere in the system, and the bias *p* of the Boolean functions is *p* = 0.5. With fully random functions the network operates in the critical regime. Figure 4(a) corresponds to the addition and Figure 4(b) to the removal of canalizing functions. In both cases we used networks with *N* = 1481 (the same number of genes as in the *E. coli* network). In a homogeneous random network with *N* genes and connectivity *K*, just by chance there are *N_c_* = *N*×*P_c_*(*K*) genes regulated by canalizing functions, and *N_nc_* = *N*×(1−*P_c_*(*K*)) genes regulated by non-canalizing functions. By forcing a fraction *q* of these *N_nc_* genes to be regulated by canalizing functions, the overall fraction *f_c_* of canalizing functions in the network increases according to *f_c_* = (*N_c_*+*q*×*N_nc_*)/*N*. Analogously, by removing a fraction *q* of the *N_c_* canalizing functions present just by chance, the fraction *f_c_* of remaining canalizing functions is *f_c_* = *N_c_*(1−*q*). In Figures 4(a) and 4(b) we present results for *q* = 0.1, 0.3, 0.5, 0.7 and 0.9, which produce the corresponding values of *f_c_* displayed in the Figure. As we can see from Figure 4, for homogeneous random networks the addition or removal of even a small fraction of canalizing functions, on top of the ones that are already present by chance, has a much bigger effect on the dynamics than for the *E. coli* network.

These results strongly suggest that the critical behavior observed in the dynamics of the genetic networks of real organisms, and its robustness to changes in the fraction of canalizing functions, are most probably due to the network architecture rather than to the logical rules that regulate the expression of the genes. If, for instance, there were a large fraction of genes with 5 inputs in the *E. coli* network, then the presence of canalizing functions would certainly affect the dynamical regime in which the network operates. But given that most of the genes have 1, 2 or 3 inputs, there is already a large fraction of canalizing functions just by chance. Adding more does not substantially affect the dynamical behavior.

## Discussion

Our results show that, to the best of the current experimental data available, and within numerical accuracy, the Boolean dynamics of the GRN of five organisms from four different kingdoms are critical. We have used two small well documented networks for specific patterning processes in plants (*A. thaliana*) and animals (*D. melanogaster*), for which both the topology and the Boolean functions are known and correspond to thoroughly studied processes at the molecular level. We also tested larger GRN for unicellular organisms (*E. coli*, *B. subtilis* and *S. cerevisiae*) in which the logical rules are not known. Thus, in the absence of other knowledge, we used random regulatory phrases constructed with gene expression probabilities inferred from microarray experiments. How ever, the results are essentially the same when the fraction of canalizing functions with the same gene expression probabilities varies considerably. The fact that all these GRN, constructed with completely different approaches, for distinct organisms and of different sizes exhibit critical Boolean dynamics is of outmost interest and strongly suggest that this might be a generic characteristic with far reaching consequences. For there is evidence that criticality confers clear evolutionary advantages to living organisms, because it is only close to criticality that robustness and adaptability can coexist. It remains an open problem to determine how criticality has emerged throughout evolution, i.e. to devise biologically relevant models of network growth that generate critical dynamics. Such models must take into account not only the evolution of the network topology, but also the emergence of the regulatory phrases through which the genes interact. If this critical behavior is corroborated as more and better experimental data become available, and with more detailed dynamic models, criticality at the genetic level may become a fundamental evolutionary mechanism that renders the stability and diversity that we observe in living organisms.

## Materials and Methods

### Phase transition in networks with realistic topology

We computed the phase transition displayed in Fig. 2(b) by implementing the Boolean dynamics in networks with scale-free output topology and Poisson input topology. Such networks are easily generated in the computer by firs assigning to each gene *g_n_* its number of outputs *l_n_*, taken from a scale-free probability distribution *P_o_*(*l*) = *Cl* ^−γ^, where *C* is the normalization constant and γ is the scale-free exponent. Once every gene has been assigned with a number of outputs, the *l_n_* outputs of each gene *g_n_* are chosen randomly from anywhere in the network. By this process, the inputs of each gene are automatically set with a Poisson distribution 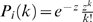 whose average *z* depends on the scale free exponent γ. There is a fraction *P_i_*(0) = *e* ^−*z*^ of genes which do not have inputs and hence remain frozen throughout the temporal evolution of the system. Those genes were not perturbed. Only genes with a nonzero number of inputs were perturbed. We used networks with *N* = 1000 genes since this is the order of magnitude of the gene transcription networks available in the databases. We run the dynamics for 1000 time steps, starting out from two initial conditions differing in 20 genes (2%). After these 1000 time steps we computed the Hamming distance between the resulting states. Each point in Fig. 2(b) is the average of this Hamming distance over 10000 different pairs of initial conditions.

Once we know the regulators of every gene in the network, the Boolean functions are assigned as follows. A Boolean function of *k* inputs has 2*^k^* values, one for each of the 2*^k^* configurations of the *k* inputs. For each of these 2*^k^* configurations we generate a random number *z* uniformly distributed in the interval [0,1]. If *z*≤*p* we set the value of the Boolean function equal to 1 for the corresponding configuration of the inputs. If *z*>*p*, we set the value of the Boolean function equal to 0. We repeat this process for all the configurations of the Boolean function and for all the Boolean functions in the network. In this algorithm the parameter *p* is the gene expression probability inferred from microarray experiments.

### Microarray Data

For *E. coli* all available microarray experiments in the Stanford Microarray Database (SMD, see http://genome-www5.stanford.edu) were incorporated in the inference algorithm, except by experiments that increased the number of false positives (see Assessing Inference Success below); 107 experiments were selected in total from SMD. Also, 45 microarray experiments were incorporated from Gosset et al, 2004 and Zhang et al, 2005. For *B. subtilis* all the available microarray experiments in the KEGG Expression Database (www.genome.jp/kegg/expression) were incorporated except experiments that increased the number of false positives; 69 experiments in total. For *S. cerevisiae* basically all the data form the three experimenters were retrieved from SMD. The name of the experimenter and the number of microarrays are as follows: Gasch, 138; DeRisi, 29; Spellman, 56. Only the cell cycle experiments from Spellman were rejected because they do not conform to the hypothesis of the Bayesian inference algorithm, which requires the absence of oscillating variables. See the Supporting Information (Text S1) for a complete relation of ID's of the incorporated experiments for the three organisms.

### Data Normalization

The data were retrieved from SMD as Log Ratios (base 2). These data were already background corrected and mean normalized by SMD itself. Only features with no flags were selected. Data from (Gosset et al, 2004; Zhang et al, 2005) were background corrected and normalized by Affymetrix Microarray Suite 5.0. Log Ratios (base 2) were then calculated between the wild type experiments and the mutants with and without glucose. The data from the KEGG Expression Database were already normalized when retrieved. They were only background corrected by us; Log ratios (base 2) were obtained.

### Inference Algorithm

Only Log Ratios greater than a certain threshold *T*_0_ were considered (see Assessing Inference Success). With the filtered data we performed standard Bayesian parametric inference with variables of two states, inhibited (repressed) and induced (activated). The Equivalent Sample Size was set equal to 4. The induction or repression of a gene were established if the average of the *a posteriori* distribution was equal to or greater than 70% for induction, and equal to or lower than 30% for repression. A detailed example of the inference algorithm is presented below.

### Assessing inference success

The nature of the regulation (activation or repression) of many of the established regulations in the networks of *E. coli* and *B. subtilis* is reported in the corresponding databases (Salgado et al, 2006; Makita et al, 2004). From this information it is possible to generate regulatory phrases only for those genes with one regulator. Thus we compared the one-regulator phrases already reported with our inferred one-regulator phrases. The inference success was established as the percentage of coincidences between our inferred phrases and the reported ones. The inference success increases as the threshold *T*_0_ increases. However, the total number of phrases that can be inferred diminishes as *T*_0_ increases. A good compromise between low percentage of false positives (high inference success) and a good statistics was achieved at *T*_0_ = 1.50 for *E. coli*, and *T*_0_ = 1.30 for *B. subtilis*. From these results we found that a good threshold for *S. cerevisiae*, for which the nature of the regulations is not reported, is *T*_0_ = 1.50. Note that a threshold of 1.5 in Log_2_ Ratios is equivalent to almost a three fold change in expression intensities.

### Example of inference

We illustrate the inference technique to obtain the value of the parameter *p* from microarray experiments with a specific example. Suppose that gene A is regulated by genes B and C, and that we want to determine the regulatory phrases, i.e. how A changes its expression due to the joint combinatorial changes of B and C. In order to do so, we need a set of microarray experiments in different conditions that have been already normalized and their background noise subtracted. We use the data shown in Table 4 for the gene expression level of three genes (log ratios base 2), obtained from two color spotted microarrays. In this table, “Cond_i_” refers to one microarray experiment in the i^th^ condition (not necessarily all the conditions have to be different). For instance, Cond_1_ may be the wild type condition, whereas Cond_2_ may be a condition in which a global regulator has been knocked out. In general, the level of expression of a given gene is different from one condition to another. Namely, there may be over expressions or under expressions across the different conditions. To filter out only the biggest changes in the level of expression of the genes we use a threshold *T*_0_, and indicate the positive changes (those greater than the threshold) with an arrow pointing upwards. Analogously, we indicate the negative changes (those lower than the threshold) with an arrow pointing downwards. When no change is detected we use the symbol (—). Using the data shown in Table 4 and setting the threshold value to *T*_0_ = 1.5, we get the discrete representation shown in Table 5.

**Table 4:** Gene expression data.

	Cond_1_	Cond_2_	Cond_3_	Cond_4_	Cond_5_	Cond_6_	Cond_7_
**A**	2.34	1.56	2.05	2.56	0.65	−3.45	−2.55
**B**	1.76	1.95	2.86	2.67	−1.89	−2.06	−1.79
**C**	3.36	1.45	1.35	1.97	−1.78	−1.67	−1.99

Typical example of the gene expression data obtained from microarray experiments. Each number represents the change (increase or decrease) of the gene expression level of the corresponding gene in Condition *i* (Cond_i_), relative to its level of expression in a given reference condition. This change is measure as a log ratio in base 2. Thus, a positive number corresponds to an increase in the level of expression, whereas a negative number represents a decrease.

**Table 5:** Discretization of the gene expression change.

	Cond_1_	Cond_2_	Cond_3_	Cond_4_	Cond_5_	Cond_6_	Cond_7_
**A**	↑	↑	↑	↑	—	↓	↓
**B**	↑	↑	↑	↑	↓	↓	↓
**C**	↑	—	—	↑	↓	↓	↓

If the change of the level of expression reported in Table 4 is larger than a given threshold *T*_0_, we write an arrow pointing upwards (↑), whereas if the change of the level of expression is smaller than −*T*_0_ then we write an arrow pointing downwards (↓). The symbol (—) indicates that no significant change was detected. In this example we used a threshold *T*_0_ = 1.5 to discretize the numbers given in Table 4.

By counting how many times A changes for the different combinations of B and C given in Table 5, we obtain the data shown in Table 6. Some table entries are equal to zero, indicating that there are no data for that particular combination of B and C. Note that we have considered all the cases with three symbols (↓,—,↑), and two regulators (B and C). Now we reduce Table 6 by considering only the entries where a change can be detected (i.e., we eliminate all entries with “—”). This gives the results displayed in Table 7. For reasons that will be clear in a moment, it is necessary to add one unit of *a priori* evidence to every entry of Table 7. After this unit has been added, we obtain Table 8. Observe from this last table that, for the first combinatorial change of B and C (first row), there are three evidences where the level of expression of A decreases and one in which it increases. If these were all the evidences, we could say that from 100% of the cases (3+1), in 75% of them the expression level of A increases and in 25% of them it decreases. Thus, the *a priori* evidence that we have added has the effect of changing the importance the *a posteriori* evidence. There are several ways of distributing the *a priori* evidence; in this work we have used an Equivalent Sample Size *S* = 4, which means that the 4 units of *a priori* evidence are distributed equally among all the possibilities.

**Table 6:** Occurrence count of evidences.

B	C	A		
		↓	—	↑
↓	↓	2	1	0
—	↓	0	0	0
↑	↓	0	0	0
↓	—	1	0	0
—	—	0	0	0
↑	—	0	1	2
↓	↑	0	0	0
—	↑	0	0	0
↑	↑	0	0	2

Counting of how many times A changes positively (↑), negatively (↓), or it does not change (—) for every combined instance of changes of B and C, according to the results displayed in Table 5.

**Table 7:** *A priori* and *a posteriori* evidence I.

B	C	A	
		↓	↑
↓	↓	2	0
↑	↓	0	0
↓	↑	0	0
↑	↑	0	2

After removing all the instances in Table 6 where there is no change, we end up with the data shown in this table. Note that there are some configurations of B and C for which there is no evidence for a corresponding change in A.

**Table 8:** *A priori* and *a posteriori* evidence II.

B	C	A	
		↓	↑
↓	↓	3	1
↑	↓	1	1
↓	↑	1	1
↑	↑	1	3

To every instance of A shown in the previous table, we add an *a priori* evidence of 1, which results in the data displayed here. This has the effect of changing the importance of the *a posteriori* evidence.

Finally, given the *a posteriori* evidence, in order to decide whether the expression level of A increases or decreases we use a second threshold *T*_1_. If the percentage of *a posteriori* evidence is greater or equal than *T*_1_, then a regulatory phrase has been established. Using a threshold *T*_1_ = 75% we obtain the results shown in Table 9. We have used the symbol “—” to state that the evidence does not support a decision. To infer the regulatory phrases from the microarray experiments analyzed in this work we used the value *T*_1_ = 75%.

**Table 9:** Inferred table of regulation.

B	C	A
↓	↓	↓
↑	↓	—
↓	↑	—
↑	↑	↑

With a threshold *T*_1_ = 75% for the a posteriori evidence in Table 8, we inferred the most probable effect of the combined changes of B and C on the expression of A. With the information available, only two regulatory phrases could be inferred in this example.

Once a set of phrases has been successfully established, we determine the probability of gene expression as the quotient of activatory phrases between the total of inferred phrases. In the case shown in Table 9, two phrases have been successfully established, one activatory and the other inhibitory. Thus, the estimated probability of gene expression in this example would be *p* = ½.

To validate our methodology for inferring regulatory phrases we use the nature of the regulation already reported in the data bases for the case of simple regulations, namely, when only one regulator determines the expression of the regulated gene (gene A is only regulated by gene B). For example, in *E. coli* the gene *alaW* is regulated (positively) only by *fis*. In the Regulon Data Base this regulation is represented as:
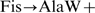
The plus sign at the end indicates that *alaW* is positively retulated by *fis*. The table with the two phrases follows immediately:

*fis* *alaW*

↓ ↓

↑ ↑

**Figure 5:**
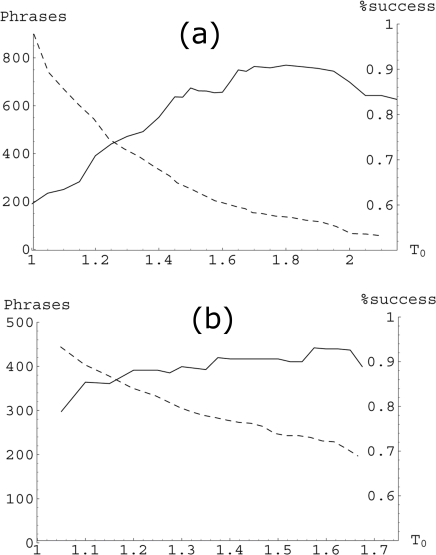
Inference success. This figure shows the compromise between the inference success (solid line), and the total number of inferred phrases (dashed line) as functions of the threshold *T*_0_ for (a) *E. coli* and (b) *B. subtilis*. Note that increasing the inference success decreases the number of inferred phrases. A compromise has to be established by adequately choosing *T*_0_. We have chosen *T*_0_ = 1.5 for *E. coli* and *T*_0_ = 1.3 for *B. subtilis*.

Note that this information in the RegulonDB cannot be used to validate our methodology for genes with more than one regulator. This is because the regulation is combinatorial. To validate our methodology we compared only the inferred phrases for simple regulations with the ones reported in the RegulonDB or the DBTBS. Figure 5 shows the inference success (the fraction of regulatory phrases form simple regulations that matched the phrases obtained from the curated databases) as a function of the threshold *T*_0_. In the same graphs we have plotted the total number of inferred phrases (including the phrases from simple regulations). As we can see, the inference becomes better as the threshold *T*_0_ increases. However, the total number of inferred phrases decreases with the threshold. In order to have a good statistics (more than 250 inferred phrases) and a high inference success (around 90%), in this work we have chosen *T*_0_ = 1.5 in *E. coli* and *T*_0_ = 1.3 in *B. subtilis*.

## Supporting Information

Text S1 Java Applet of the dynamics and list of ID numbers for the microarray experiments used in this work.
(0.05 MB DOC)
