# Supplementary material for: Critical Dynamics in Genetic Regulatory Networks: Examples from Four Kingdoms
Source: PLoS One. 2008 Jun 18;3(6):e2456. doi: 10.1371/journal.pone.0002456 (PMC2423472; doi:10.1371/journal.pone.0002456)
Supplement: Text S1 — Java Applet of the dynamics and list of ID numbers for the microarray experiments used in this work. (0.05 MB DOC) [file pone.0002456.s001.doc]

# Supporting Information for the manuscript

Critical Dynamics in Genetic Regulatory Networks: Examples from Four Kingdoms

**Boolean dynamics**

A Java applet showing the animation of the propagation of perturbations in Boolean networks operating in the three different dynamical regimes (ordered, critical and chaotic) can be found at

[**http://www.fis.unam.mx/~max/criticality/animation.html**](http://www.fis.unam.mx/~max/criticality/animation.html)

**Identification numbers of the microarray experiments used in this work.**

Relation of ID’s of the incorporated microarrays in the parametric inference of regulatory phrases for *B. subtilis*. The date were downloaded from <http://www.genome.jp/kegg/expression>

ex0000263

ex0000264

ex0000265

ex0000266

ex0000267

ex0000268

ex0000269

ex0000270

ex0000271

ex0000272

ex0000273

ex0000274

ex0000275

ex0000276

ex0000277

ex0000278

ex0000279

ex0000280

ex0000281

ex0000282

ex0000283

ex0000284

ex0000285

ex0000286

ex0000260

ex0000261

ex0000262

ex0000259

ex0000258

ex0000358

ex0000369

ex0000370

ex0001750

ex0001751

ex0001752

ex0001753

ex0000824

ex0001597

ex0001598

ex0001599

ex0001600

ex0001437

ex0001439

ex0001440

ex0000798

ex0000360

ex0000940

ex0000941

ex0000942

ex0000943

ex0000944

ex0000945

ex0000340

ex0000377

ex0000782

ex0000381

ex0001360

ex0000659

ex0000660

ex0000661

ex0000744

ex0000746

ex0000745

ex0000747

ex0000749

ex0000758

ex0000748

ex0000785

ex0000395

**Supplementary Data 2**

The following ID’s correspond to microarray experiments downloaded from The Stanford Microarray Database (SMD) (<http://genome-www5.stanford.edu/>). The microarray data were incorporated in the parametric inference of regulatory phrases of *E. coli*. The data were retrieved as Log Ratios (base 2) and background corrected and mean normalized by SMD itself. Only features with no flags were selected.

Experimenter ID: JONB

8377

8379

8536

25831

15343

13838

15341

15342

13840

15337

15338

15336

Experimenter ID: KHODURSK

5265

5277

5278

5268

5281

5272

5273

1642

1646

1644

1647

1649

1643

1650

1285

1290

1292

1911

1908

1912

1913

1914

1909

1915

1916

14832

14830

14831

14829

1596

2353

1598

2354

8457

8458

8459

1601

1604

1608

1599

2357

2355

1602

1606

1610

1600

2358

1603

1611

9152

Experimenter ID: KANGSEOK

32748

32749

32746

32757

32759

32760

Experimenter ID: CHRISM

13076

13077

36049

36051

36054

Experimenter ID: MBSUE

19887

19888

19627

19628

19629

18991

18989

19343

19344

19345

18990

19886

19807

19810

19812

Other 45 experiments with results published in Refs. 9 and 32 of the main text were obtained upon request to the authors.

**Supplementary Data 3**

The following ID’s correspond to microarray experiments downloaded from The Stanford Microarray Database (SMD) (<http://genome-www5.stanford.edu/>). The microarray data were incorporated in the parametric inference of regulatory phrases of *S. cerevisiae*. The data were retrieved as Log Ratios (base 2), background corrected and mean normalized by SMD itself. Only features with no flags were selected.

Experimenter ID: AGASCH

7529

7530

4779

691

4778

12801

4780

4781

4782

7528

692

989

990

991

992

985

986

6377

987

988

993

6679

6681

6685

6688

6668

6672

6676

972

975

963

964

961

962

1661

974

971

2558

2556

2064

2557

2554

2555

830

831

834

836

5357

838

840

842

5245

5243

5246

5244

5239

5237

5240

5242

984

1250

1257

1251

1252

1253

1249

1254

1255

977

983

978

979

980

976

981

982

8525

6364

6439

8522

6362

8523

8524

8520

6349

6351

6344

6357

6358

6354

8521

8528

8526

825

1108

1106

824

1155

819

1139

1104

827

815

817

812

5358

4872

4874

7547

7549

7535

7536

7537

7538

7539

4785

4787

4786

2560

2559

4784

4783

4789

809

814

1258

6361

811

810

813

7540

4047

4048

7541

7542

877

878

5187

Experimenter ID: SPELLMAN

686

684

681

680

1686

685

512

513

514

515

516

517

518

519

8227

8228

8229

8230

8233

8234

8235

584

585

597

598

599

8193

8217

8195

8197

8204

8210

8213

8247

497

781

797

799

800

801

802

106

107

108

12744

109

115

410

409

408

407

406

2144

116

117

118

Experimenter ID: JDERISI

1303

1311

1312

1302

1309

1313

1310

8282

8284

8287

8286

8291

7998

7999

8000

8003

7893

7895

6854

6859

6824

6841

6826

6856

6834

6836

6839

7985

8289
